# Ccr7 null mice are protected against diet-induced obesity via Ucp1 upregulation and enhanced energy expenditure

**DOI:** 10.1186/s12986-019-0372-5

**Published:** 2019-07-04

**Authors:** Tomomi Sano, Taiki Sanada, Yusuke Sotomaru, Takanori Shinjo, Misaki Iwashita, Akiko Yamashita, Takao Fukuda, Terukazu Sanui, Tomoichiro Asano, Takashi Kanematsu, Fusanori Nishimura

**Affiliations:** 1 Section of Periodontology, Kyushu University Faculty of Dental Science, 3-1-1 Maidashi, Higashi-ku, Fukuoka, 812-8582 Japan; 2 Natural Science Center for Basic Research and Development, Hiroshima University, Hiroshima, Japan; 3 Section of Vascular Cell Biology, Joslin Diabetes Center, Harvard Medical School, Boston, MA USA; 4 Department of Biological Chemistry, Hiroshima University Institute of Biomedical and Health Sciences, Hiroshima, Japan; 5 Department of Cellular and Molecular Pharmacology, Hiroshima University Institute of Biomedical and Health Sciences, Hiroshima, Japan

**Keywords:** Obesity, Energy expenditure, Brown adipose tissue, Adipose tissue, Inflammation

## Abstract

**Background:**

The chemokine receptor CCR7, expressed on various immune cells, is associated with cell migration and lympho-node homing. Mice lacking Ccr7 are protected from diet-induced obesity and subsequent insulin resistance. We evaluated the mechanism underlying these protective effects from the standpoint of energy expenditure.

**Methods:**

Wild-type and Ccr7 null mice were fed a high-fat diet, and the regulation of energy metabolism and energy metabolism-related molecules, e.g., Ucp1, *Cidea*, and *Pgc1α*, were evaluated.

**Results:**

Food intake did not differ between groups. O_2_ consumption and CO_2_ production were higher in Ccr7 null mice than in wild-type mice, despite a similar respiratory quotient and glucose and lipid utilization, suggesting that energy expenditure increased in Ccr7 null mice via enhanced metabolism. In white adipose tissues of Ccr7 null mice, *Prdm16*, *Cd137*, *Tmem26*, *Th*, and *Tbx1* expression increased. Similarly, in brown adipose tissues of Ccr7 null mice, *Dio2*, *Pgc1α*, *Cidea*, *Sirt1*, and *Adiponectin* expression increased. In both white and brown adipose tissues, Ucp1 gene and protein expression levels were higher in null mice than in wild-type mice.

**Conclusions:**

In Ccr7 null mice, browning of white adipocytes as well as the activation of brown adipocytes cause enhanced energy metabolism, resulting in protection against diet-induced obesity.

**Electronic supplementary material:**

The online version of this article (10.1186/s12986-019-0372-5) contains supplementary material, which is available to authorized users.

## Background

It is well accepted that obesity increases the risk of other related complications such as insulin resistance and atherosclerosis. It is also suggested that obesity-induced inflammatory changes accompanied by the infiltration of immune cells into adipose tissue play crucial roles in the establishment of such complications. The number of obese subjects is still increasing in many parts of the world due possibly to the rapid economic changes, and, thus, the development of new and effective therapeutic strategy against obesity and/or insulin resistance is still urgently needed. We believe that adipose tissue browning is one such strategy against obesity.

Adipose tissues are classified into white (WAT) and brown adipose tissue (BAT). The majority of adipose tissues in adults are believed to be WAT, with abundant triglycerides in adipocytes. BAT utilizes lipids as a substrate and produces heat. The volume of BAT is greater in neonatal infants than in adults, and individual differences in the rate of decline and activity are associated with the development of obesity [1]. Thus, the activation of BAT is a therapeutic target for obesity.

Heat production in BAT depends on the activity of uncoupling protein 1 (UCP1), a proton channel involved in uncoupling ATP synthesis, located in the inner membrane of mitochondria. It produces heat in response to various stimuli [2]. The loss of function of Ucp1 in mice accelerates the development of obesity in response to a high-fat diet [3], while the overexpression of Ucp1 in WAT results in protection against diet-induced obesity in mouse models [4]. In WAT, brown adipocytes, also called brite or beige adipocytes, are observed. In WATs of KK-Ay mice fed water containing acetate for 16 weeks, beige-related gene expression is upregulated [5]. Since beige adipocytes also produce heat, similar to brown adipocytes, and enhance energy expenditure, conversion from white adipocytes to beige adipocytes is a particularly interesting subject for the development of therapeutic strategies against obesity.

CCR7 is expressed on the surfaces of dendritic cells and certain T- and B-lymphocytes. It acts as a chemokine receptor for CCL19. CCR7 is also expressed on inflammatory M1 but not anti-inflammatory M2 macrophages. We previously reported that Ccl19 expression is markedly up-regulated in adipocytes co-cultured with macrophages in the presence of toll like receptor-4 ligand, a bacterial endotoxin. In fact, the Ccl19 concentrations in the sera of ob/ob and high-fat diet-induced obese mice increase dramatically following endotoxin infusion via the tail vein. Furthermore, mice lacking Ccr7 are protected from diet-induced obesity. In these mice, we also observed the adipose tissue expression of adiponectin, liver expression of *AdipoR1* and *AdopoR2*, as well as heat production in cold conditions [6]. These findings are supported by studies by other groups [7], but opposing results have also been obtained [8]. We hypothesized that Ccr7 may play important role in browning process as loss of function of Ccr7 results in the suppression of the development of obesity and/or insulin resistance. CCR7 is a chemokine receptor for many immune cells as mentioned. Therefore, in this study, to verify our previous results, we aimed to see the biological relationship between immune cell recruitment into the adipose tissue by Ccr7 and metabolic regulation system of energy expenditure.

## Methods

### Generation of Ccr7 null mice

Ccr7 null mice were a generous gift from Dr. Martin Lipp at the Department of Tumor Genetics and Immunogenetics, Max-Delbruck-Center of Molecular Medicine, Germany. The generation of Ccr7 null mice was explained in detail in a previous study; mice were kept under specific pathogen-free conditions until use [9].

### Animals

Male Ccr7 null (KO) mice (*n* = 6) and wild-type (WT) C57BL/6 J mice (n = 6) were housed under specific pathogen-free conditions at the Kasumi Laboratory Animal Center of Hiroshima University under a 12-h light/dark cycle at 23 ± 2 °C. KO and WT mice were not from the same colony. Mice were fed a high-fat diet (HFD; 60.7% fat, 17.9% protein, 21.4% nitrogen free extracts; Oriental Yeast, Tokyo) from 8 weeks of age, and male mice were used in all experiments. This study was approved by the Animal Care and Use Committee of Hiroshima University (permission number: A15–81) and was performed in accordance with the Guide for Hiroshima University Animal Experimentation Regulation. Body weights were monitored and food intake was recorded by subtracting the amount of food left in the cage from the daily amount given, at 2 p.m. (day) and 2 a.m. (night). At 20 weeks of age, the mice were sacrificed.

### Locomotor activity analysis

A locomotor activity analysis was performed according to previously reported methods [10]. Locomotor activity was recorded using a video camera (HDR-XR550V; Sony, Tokyo, Japan) and results were analyzed using the ANY-maze video tracking system (Stoelting Co., Wood Dale, IL, USA). Mice fed an HFD were maintained in a cage (a square arena, 30 cm × 30 cm, with 40-cm high opaque walls) for 1 week before 18 weeks of age, and the total distance traveled was measured for 24 h.

### Analysis of energy metabolism

Nineteen-week-old WT and KO mice fed the HFD were subjected to metabolic analyses. Oxygen consumption (VO_2_) and carbon dioxide production (VCO_2_) were measured using a computer-controlled open-circuit calorimetry system (Oxymax System; Columbus Instruments, Columbus, OH, USA). All mice were acclimatized for 24 h before measurements, and VO_2_ and VCO_2_ data were recorded for 2 days. The respiratory exchange ratio was calculated as the ratio of VCO_2_ to VO_2_. Energy expenditure was calculated using the following equation: heat = CV × VO_2_, where CV = 3.815 + 1.232 × respiratory exchange ratio (CV, calorific value based on the observed respiratory exchange ratio).

### Histochemistry

Epididymal and inguinal WATs (eWAT and iWAT, respectively), BAT, and the muscle were fixed with 4% paraformaldehyde phosphate buffer solution and embedded in paraffin. Muscle and iWAT samples were subjected to standard hematoxylin-eosin staining [10]. Cell areas were measured using ImageJ. Immunohistochemical staining of adipose tissue was performed using paraffin sections with F4/80 (mouse macrophage and microglial marker), Cd11b (mouse macrophage and microglial marker), and Ucp1 antibodies.

### Quantitative real-time PCR

Quantitative Real-time PCR was performed according to the same methods reported previously [11]. cDNA was constructed using the ReverTra Ace qPCR RT Kit (Toyobo, Tokyo, Japan). The real-time PCR was performed using the KAPA SYBR FAST qPCR Kit (ABI Prism, Applied Biosystems, Foster city, CA), and the reactions were conducted on the 7300 Real-Time PCR system (Applied Biosystems, Foster city, CA). The mRNA expression levels of *Ucp1, Cidea, Pgc1a, Prdm16, Cd137, Tmem26, Th*, and *Tbx1* in epididymal adipose tissues were quantified, and the levels of *Ucp1, Dio2, Pgc1a, Cidea, Sirt1*, and *Adiponectin* in brown adipose tissues were quantified. The primer sequences are listed in Table 1. Data were normalized against *Gapdh* levels unless specified and were calculated as fold change values relative to expression in control WT mice. In some experiments, the data were also normalized against *β-actin* levels for better confirmation.

**Table 1:** Primers used in the study

Gene	Forward primer	Reverse primer
*Gapdh*	AATGTGTCCGTCGTGGATCTGA	GATGCCTGCTTCACCACCTTCT
*Ucp1*	CACCTTCCCGCTGGACACT	CCCTAGGACACCTTTATACCTAATGG
*Cidea*	ATCACAACTGGCCTGGTTACG	TACTACCCGGTGTCCATTTCT
*Pgc1α*	AGCCGTGACCACTGACAACGAG	GCTGCATGGTTCTGAGTGCTAA
*Prdm16*	CAGCACGGTGAAGCCATTC	GCGTGCATCCGCTTGTG
*Cd137*	GGCCGTTTAGGAAAGGGACA	CAGGAGTCATGCAGAGGCAA
*Tmem26*	GAAACCAGTATTGCAGCACCC	CCCATTCCATTGGTGGCTCT
*Th*	CCTGGAGTACTTTGTGCGCT	GGGAACCAGGGAACCTTGTC
*Tbx1*	CCGGTGAAGAAGAACCCGAA	AACATTCGTCTGCCTGCCTT
*Dio2*	CAGTGTGGTGCACGTCTCCAATC	TGAACCAAAGTTGACCACCAG
*Sirt1*	GCATAGATACCGTCTCTTGATCTGAA	TGTGAAGTTACTGCAGGAGTGTAAA
*Adiponectin*	TGTTCCTCTTAATCCTGCCCA	CCAACCTGCACAAGTTCCCTT
*β-actin*	TCTTTGATGTCACGCACGAT	TACAGCTTCACCACCACA

### Immunoblot assay

BAT and eWAT were homogenized in a lysis buffer containing 50 mm Tris-HCl, pH 7.4, 150 mm NaCl, 1 mm NaF, 1 mm EDTA, 1 mm EGTA, 1% Triton X-100, 500 μM Na_3_VO_4_, and protease inhibitors. After 30 min of incubation on ice, cell lysates were centrifuged at 20,000×*g* for 30 min, and supernatants were collected. The protein concentration was measured using the Protein Assay Rapid Kit (Wako, Osaka, Japan), and the lysate samples were used for immunoblotting. The primary antibodies were as follows: anti-Gapdh antibody (Proteintech, Rosemount, IL, USA) and anti-Ucp1 antibody (Abcam, Cambridge, UK). For the secondary antibodies, Anti-rabbit IgG, HRP-linked Antibody (Cell Signaling, Danvers, MA, USA) was used. An Enhanced Chemiluminescence Western Detection Reagent (Nacalai Tesque) was used for signal development. Resultant signals were captured using an ImageQuant LAS 4000 Mini Detection System (GE Healthcare Life Sciences). The density of each band was analyzed using ImageJ.

### Statistical analysis

Data are expressed as means ± S.E. Statistical analyses were performed using Student’s *t*-tests within the Excel software package (Microsoft). Values of *P* < 0.05 were considered significant.

## Results

**Fig. 1:**
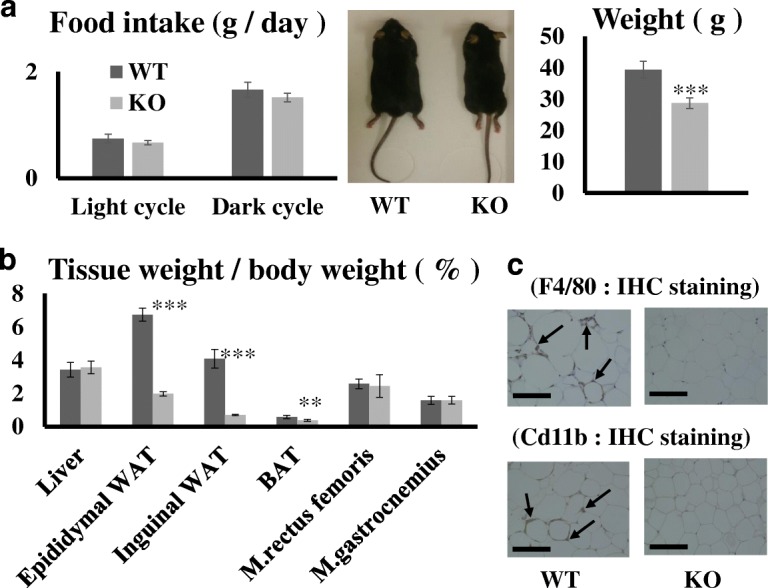
Differences in food intake and weight between C-C chemokine receptor type 7 (Ccr7) null mice and wild-type mice. **a** Food intake during light and dark cycles and body weight at 20 weeks. **b** Ratios of the weights of each tissue and organ against the total weight. **c** Immunostaining of F4/80 and Cd11b in eWAT of each mouse

Food intake in both the light and dark cycles did not differ significantly between WT and KO mice (Fig. 1a). We first confirmed the changes of body weight gain in each group as reported in our previous study [6] and performed subsequent experiments. We next evaluated the ratio of each tissue weight against the total body weight (Fig. 1b). Although the weights of liver and two types of muscle did not differ between the two mouse groups, adipose tissue weights were significantly lower (*P* values; Epididymal WAT 0.0004, Inguinal WAT 0.0014, and BAT 0.0216) in KO mice than in WT mice. KO mice fed a high-fat diet had 30% of the epididymal WAT in WT mice, 16.4% of the inguinal WAT, and 65% of the BAT. We also previously did not observe fatty liver in KO mice fed a high-fat diet. In this study, we further explored the histological features of muscle tissues. However, no apparent differences were detected in muscle tissues with respect to ectopic lipid deposition (Additional file 1: Figure S1a). In the WAT of KO mice, no apparent F4/80, Cd11b-positive cells were observed, while in WT mice, the accumulation of these positive cells was observed (Fig. 1c). In the subcutaneous fat, adipocyte size did not differ between WT and KO mice (Additional file 1: Figure S1b).

**Fig. 2:**
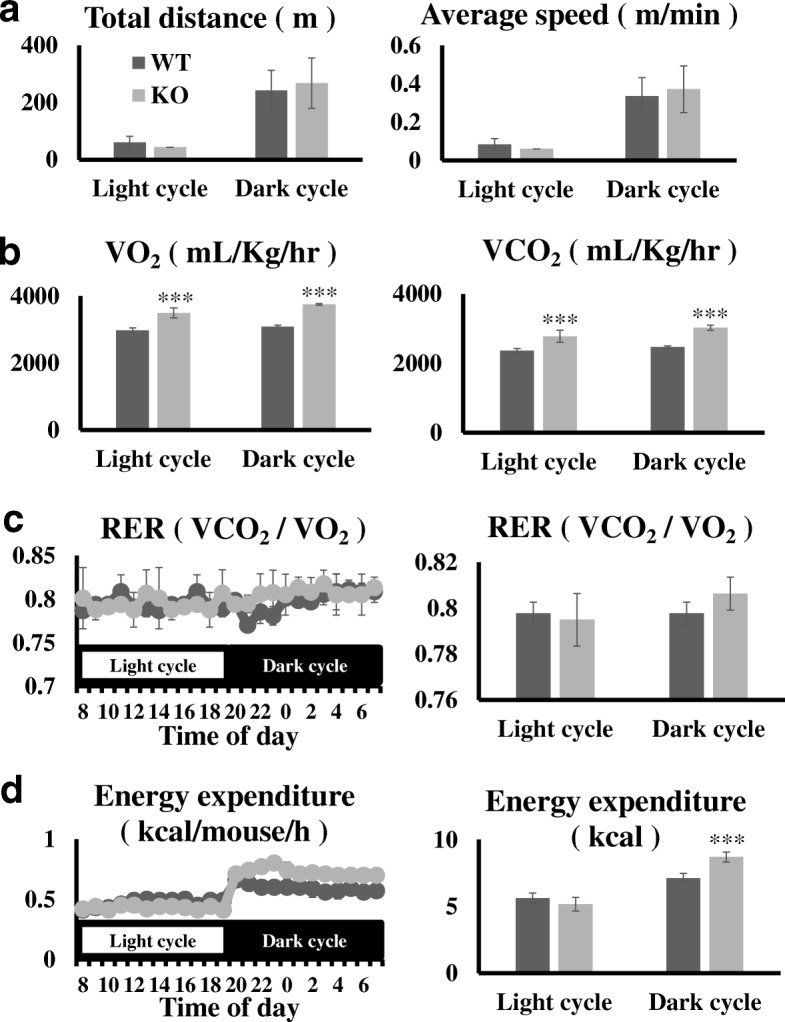
Locomotor activity and oxygen consumption in each mouse group. **a** Locomotor activity of 18-week-old wild-type (WT; HFD, *n* = 6) and Ccr7 null (KO; HFD, n = 6) mice. Total traveled distance (day, 8 a.m. to 8 p.m.; night, 8 p.m. to 8 a.m.) was analyzed using the ANY-maze video tracking system. **b** In 19-week-old WT (HFD, n = 6) and KO (HFD, n = 6) mice, oxygen consumption (VO_2_) and carbon dioxide production (VCO_2_) were assessed by an indirect calorimetric system over a 24-h period with a 12-h light/dark cycle (day from 8 a.m. to 8 p.m.); the respiratory exchange ratio (RER) **c** and energy expenditure **d** are shown. Data are presented as means ± S.E. ***, *p* < 0.001 versus the corresponding WT value

Additionally, in KO fed a high-fat diet, adipocyte maturation was less than that in WT mice fed a high-fat diet. Therefore, we assessed energy expenditure in both mice. We monitored spontaneous motor activity in both the light and dark using a 12-h cycle. However, no significant differences were observed (Fig. 2a). O_2_ consumption and CO_2_ production were significantly higher (*P* values; O_2_ light cycle 1.09E-14, dark cycle 4.99E-34, CO_2_ light cycle 1.65E-11, dark cycle 4.29E-26) in KO mice fed a high-fat diet than in WT mice fed a high-fat diet (Fig. 2b). The respiratory quotient did not differ between WT and KO mice (Fig. 2c). When we calculated energy expenditure, dark cycle energy expenditure in KO mice was significantly greater (P value; 0.0016) than that in WT mice fed a high-fat diet (Fig. 2d).

**Fig. 3:**
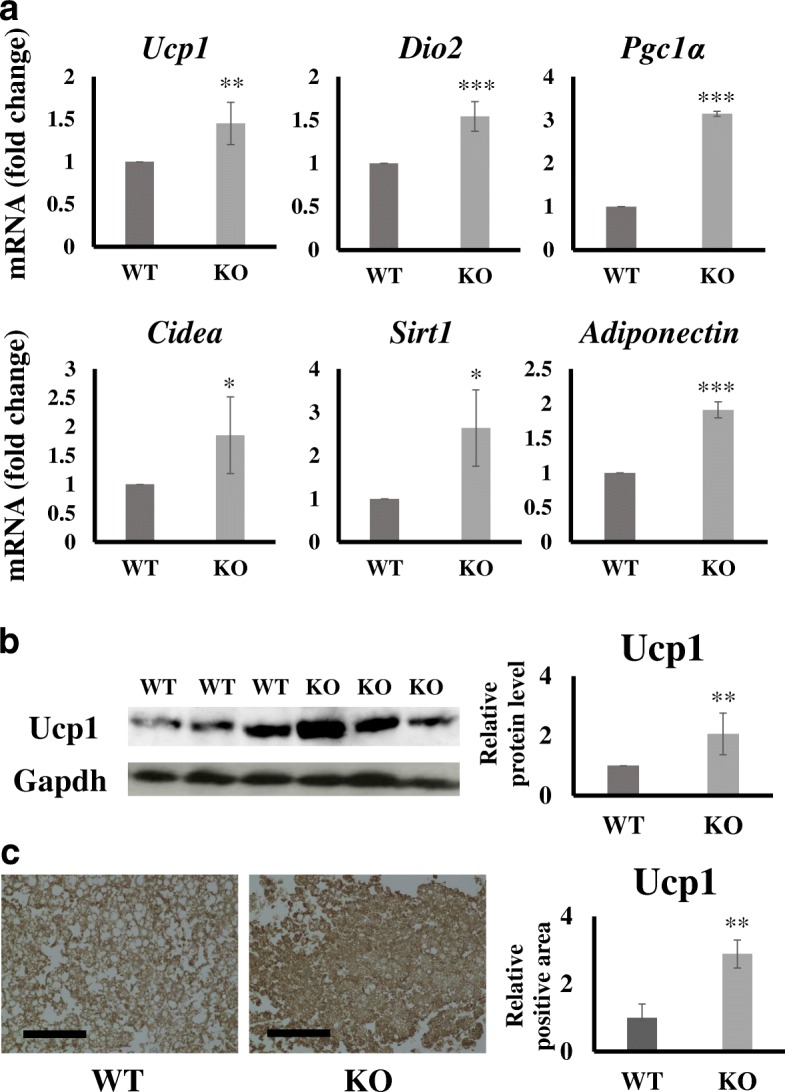
Analysis of brown adipose tissue (BAT)-related gene and protein expression levels. **a** BAT-related gene expression levels in each test group. Test mice include those fed a high-fat diet as described in the “Materials and methods” section. Gene expression was analyzed by real-time PCR. Data are normalized against *Gapdh* and expressed as fold changes relative to the expression level in WT mice. **b** Detection of Ucp1 by a western blot analysis in BAT. Western blot analyses were performed as described in the “Materials and methods” section. **c** Ucp1 staining in BAT, bar: 100 μm. Brown parts are positively stained for Ucp1. *p < 0.05, **p < 0.01 ***p < 0.001 by Student’s *t*-test

To understand the molecular basis underlying the protection of KO mice from diet-induced obesity, we compared the expression of genes related to energy expenditure in BATs of these mice. In KO mice, the expression of genes encoding *Ucp1* and other BAT markers, such as *Dio2*, *Pgc1α*, *Cidea*, *Sirt1*, and *Adiponectin* were significantly up-regulated (*P* values; *Ucp1* 3.04E-05, *Dio2* 7.30E-05, *Pgc1α* 0.0005, *Cidea* 0.0018, *Sirt1* 0.0015, and *Adiponectin* 8.94E-06) compared with levels in WT mice fed a high-fat diet (Fig. 3a, Additional file 2: Figure S2a). A western blotting analysis revealed that Ucp1 protein expression was also up-regulated in the BAT of KO mice fed a high-fat diet (Fig. 3b). Similarly, strong immunostaining of Ucp1 was observed in the BAT of KO mice (Fig. 3c).

**Fig. 4:**
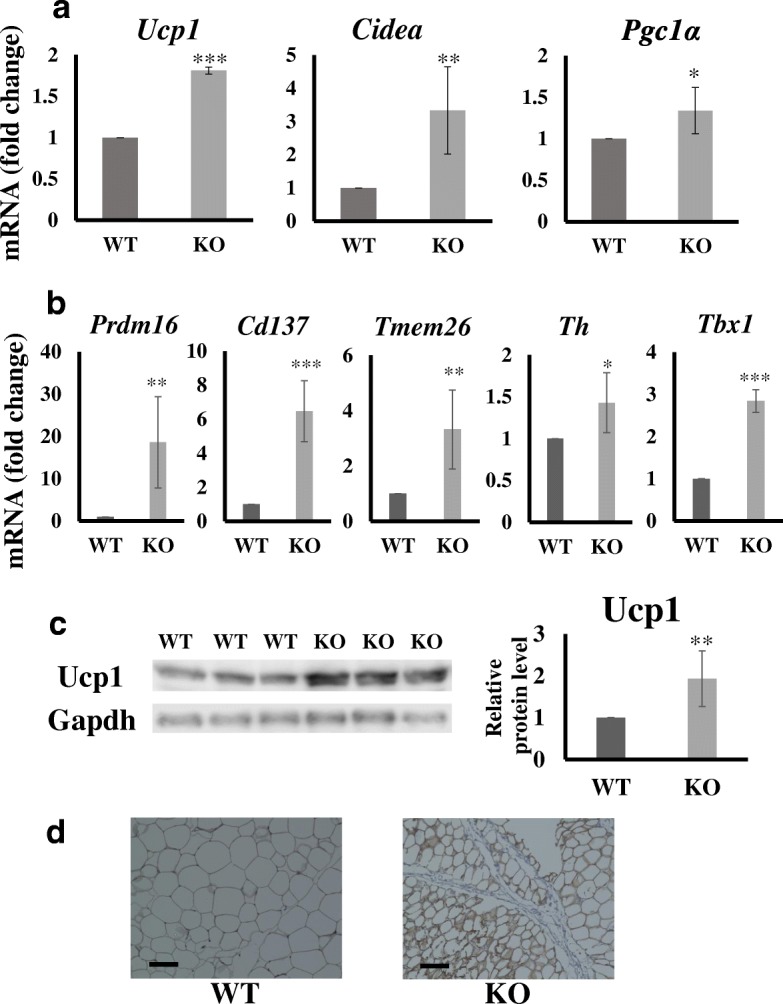
Analysis of white adipose tissue (WAT)-related gene and protein expression levels. **a**, **b** eWAT gene expression levels in each test group. Test mice include those fed a high-fat diet as described in the “Materials and methods” section. Gene expression was analyzed by real-time PCR. Data are normalized against *Gapdh* and expressed as fold changes relative to the expression level in WT mice. **c** Detection of Ucp1 by a western blot analysis in eWAT. Western blot analyses were performed as described in the “Materials and methods” section. Brown parts are positively stained for Ucp1. **d** Ucp1 staining, bar: 100 μm. *p < 0.05, **p < 0.01 ***p < 0.001 by Student’s *t*-test

We next assessed the expression of genes associated with energy expenditure in WAT. In KO mice fed a high-fat diet, the expression levels of *Ucp1*, *Cidea,* and *Pgc1α* were higher than those in WT mice (Fig. 4a, Additional file 2: Figure S2b), while in subcutaneous adipose tissue, no apparent differences in *Ucp1* were observed (Additional file 2: Figure S2c). Furthermore, in the WAT of KO mice, the expression levels of factors that promote browning, such as *Prdm18*, *Cd137*, *Teme26*, *Th*, and *Tbx1*, were significantly higher (P values; *Prdm18* 0.0012, *Cd137* 0.0003, *Tmem26* 0.0019, *Th* 0.0290, and *Tbx1* 0.0006) than those in WT mice (Fig. 4b). Ucp1 protein expression was also greater in KO mice than in WT mice (Fig. 4c); strong Ucp1 immunostaining was observed in the WAT of these mice (Fig. 4d). Ucp1 protein expression in subcutaneous adipose tissues did not differ between WT and KO mice (Additional file 2: Figure S2d). Taken together, in KO mice, adipose tissue browning is induced by a high-fat diet.

## Discussion

In this study, we started feeding HFD for WT and Ccr7 null mice at 8 weeks of age till 20 weeks. Since these mice generally reach sexual maturity at 8 weeks, we consider the mice at 8 weeks adult. And when we feed WT mice HFD, the body weight usually reaches plateau at 20 weeks. We therefore performed the experiments between 8 weeks and 20 weeks of age. We previously reported that Ccr7 null mice are protected from diet-induced obesity [6]. While WAT stores excess energy, BAT utilizes stored energy and produces heat. However, WAT acquires a BAT-like phenotype by browning and expresses BAT-specific genes, such as heat-producing genes. Ccr7 null mice fed a high-fat diet showed increased energy expenditure, the browning of WAT, as well as the activation of BAT. These factors may explain why these mice were protected from diet-induced obesity. Adipose tissue volume of both types of WAT (eWAT and iWAT) itself was smaller in Ccr7 null mice than in WT mice. However, adipocyte size was only different in eWAT. Although speculative, adipocyte browning could have suppressed hypertrophic changes in eWAT. The similar phenomenon is reported in other studies [12, 13].

Uncoupling protein 1 is localized in the inner membrane of mitochondria and uncouples ATP synthesis. UCP1 plays important roles in the maintenance of energy homeostasis and protection against obesity. Ucp1 null mice develop obesity, while the overexpression of Ucp1 in mice results in protection from diet-induced obesity [4, 14]. UCP1 homologues, i.e., UCP2 and UCP3, have similar roles and are potential therapeutic target molecules against obesity. UCP2 is ubiquitously expressed at low levels throughout the body, while UCP3 expression is limited to the skeletal muscle and cardiac muscles [15, 16]. Mice with muscle-specific overexpression of Ucp3 did not develop obesity and showed increased energy metabolism and normal glucose tolerance upon high-fat feeding [17].

In this study, we did not observe significant differences in the expression of *Ucp2* and *Ucp3* between mice (data not shown). Thus, we hypothesized that Ucp1 was the primary molecule responsible for regulating energy expenditure in Ccr7 null mice. In WT mice fed a high-fat diet, excess energy accumulates in adipose tissues and accelerates adipose tissue inflammation.

We observed Cd11b- and F4/80-positive macrophage infiltration in WT mice fed a high-fat diet but not in Ccr7 null mice fed a high-fat diet; it is possible that this can be explained by the protection against obesity and reduced inflammation in the adipose tissue via increased energy expenditure in these mice. We observed high *Sirt1* expression in Ccr7 null mice, which activates Pparγ by de-acetylation and accelerates the browning of WAT [18]. Incubation of pre-adipocytes derived from murine adipose tissues with a Pparγ agonist results in increased expression of Ucp1 at the gene and protein levels [19]. High *Pparγ* expression was also noted in our previous study [6]. Pparγ null mice are characterized by increased CD11b + CD11c + F4/80+ macrophages [20]. High *Pparγ* expression in Ccr7 null mice is accompanied by the browning of WAT and decreased infiltration of macrophages. High-fat diets are associated with an increased ratio of F4/80 + CD11c + CCR7+ macrophages [7]. Therefore, CCR7 may play an important role in regulating adipose tissue inflammation.

Cold stimulation, diet, and exercise may regulate Ucp1 expression [3, 21]. However, these factors did not influence the results of our experiments, which were performed under a normal, consistent temperature. Both mice consumed similar amounts of foods and showed similar motor activity. Ucp1 expression is induced by oxidative stress [22]. We noted increased *Pgc1α* expression in the adipose tissues of Ccr7 null mice fed a high-fat diet. PGC1α is known to elevate oxidative stress in adipocytes [23]. Oxidative stress increases Pgc1α expression and Pgc1α interacts with nuclear receptors, such as retinoid receptor Rxr and Ppar, and up-regulates mitochondrial synthesis and Ucp1 expression in white adipocytes [24, 25]. Thus, it is possible that the browning of WAT in Ccr7 null mice fed a high-fat diet is mediated by oxidative stress and subsequent Pgc1α expression. In fact, we previously performed gene expression profiling of adipocytes co-cultured with macrophages in the presence of bacterial endotoxins, and superoxide dismutase 2 (also known as mitochondrial super oxide dismutase) gene expression was markedly up-regulated in adipocytes upon endotoxin stimulation [26]. Superoxide dismutase 2 is a mitochondria-specific superoxide scavenger [27]. Thus, in the mitochondria of WT mouse adipose tissue with macrophage infiltration, the superoxide scavenging system may function as secondary factors, while the scavenging system does not function consistently in adipose tissue without macrophage infiltration. Pgc1α is a regulator of mitochondrial biogenesis. In the obese state, it is known that TNF-α, which is highly expressed in immune cells, inhibits Ucp1 expression in adipocytes [28]. Thus, in the current study, it is possible that increased TNF-α expression accompanied by immune cell infiltration suppressed Ucp1 expression in adipose tissue in co-operation with reduced mitochondrial biogenesis accompanied by lower Pgc1α expression as mentioned above in WT mice fed a high-fat diet. In fact, inflammatory macrophage infiltration is reported to suppress adipocyte browning [29]. Taken together, in obese adipose tissues where increased amounts of dendritic cells and macrophages are infiltrated, browning may be suppressed, and such obese state may further accelerate the inhibition of energy expenditure.

In human adults, brown adipose tissue was not clearly identified before. However, FDG-PET (positron emission tomography with fluorodeoxy-glucose) analyses clearly demonstrated the existence of brown adipose tissues in human adults, and heat production was confirmed in such brown adipose tissues [30]. Furthermore, exercise or cold temperature stimulation toward human healthy adults result in the increase in serum IRISIN and FGF21, and in vitro study showed that IRISIN and FGF21 up-regulated UCP1 expression and subsequent heat production [31]. Furthermore, in the human white adipose tissue, cold temperature promotes adipocytes to ectopically express UCP1 and, thus, it is suggested that browning may greatly regulate metabolism [32]. Moreover, human visceral fat is without doubt a great contributor for obesity and related complications, but can be a target for therapy by converting adipocytes into browning [33].

## Conclusions

Ccr7 null mice are protected from diet-induced obesity. The browning of WAT and activation of BAT are the primary mechanisms underlying this protection. Therefore, CCR7 is a potentially effective therapeutic target against obesity and/or related diseases.

## Additional files

Additional file 1: **Figure S1.** Summary of hematoxylin-eosin (HE) staining results. (a) HE staining (muscle). bar, 100 μm. (b) HE staining (iWAT). bar, 100 μm. (c) Inguinal adipocyte size in the test mice. (PPTX 561 kb)

Additional file 2: **Figure S2.** (a) *Ucp1* gene expression levels in BAT. (Data were normalized against *β-actin* levels.) (b) *Ucp1* gene expression levels in eWAT. (Data were normalized against *β-actin* levels.) (c) *Ucp1* gene expression levels in iWAT (Data were normalized against *Gapdh* levels). (d) Ucp1 staining in iWAT, bar: 100 μm. Brown parts are positively stained for Ucp1. (PPTX 591 kb)

## Data Availability

The datasets used and/or analysed during the current study are available from the corresponding author on reasonable request.

## Abbreviations

Cidea: Cell death activator
Dio2: iodothyronine deiodinase 2
Pgc1α: Peroxisome proliferator activated receptor gamma coactivator 1 alpha
Prdm16: PR domain containing 16
Sirt1: sirtuin 1
Tbx1: T-box 1
Th: tyrosine hydroxylase
Tmem26: Transmembrane protein 26
