# Supplementary figures and images for: Ccr7 null mice are protected against diet-induced obesity via Ucp1 upregulation and enhanced energy expenditure
Source: Nutr Metab (Lond). 2019 Jul 4;16:43. doi: 10.1186/s12986-019-0372-5 (PMC6610939; doi:10.1186/s12986-019-0372-5)

## Slide 1
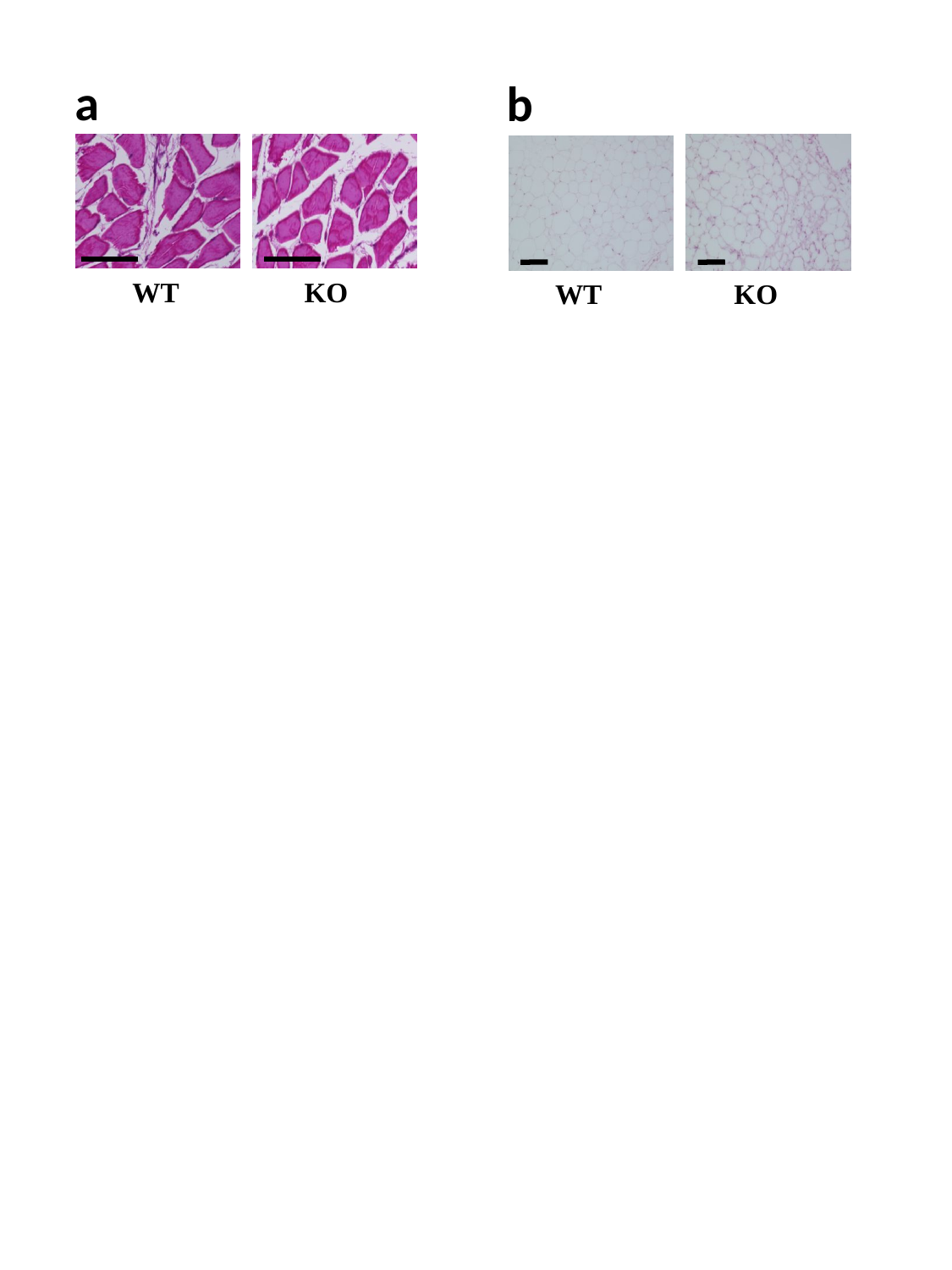

a
b
 WT KO
 WT KO

Supplement: Supplementary file 1 — Figure S1. Summary of hematoxylin-eosin (HE) staining results. (a) HE staining (muscle). bar, 100 μm. (b) HE staining (iWAT). bar, 100 μm. (c) Inguinal adipocyte size in the test mice. (PPTX 561 kb) [file 12986_2019_372_MOESM1_ESM.pptx]

## Slide 1
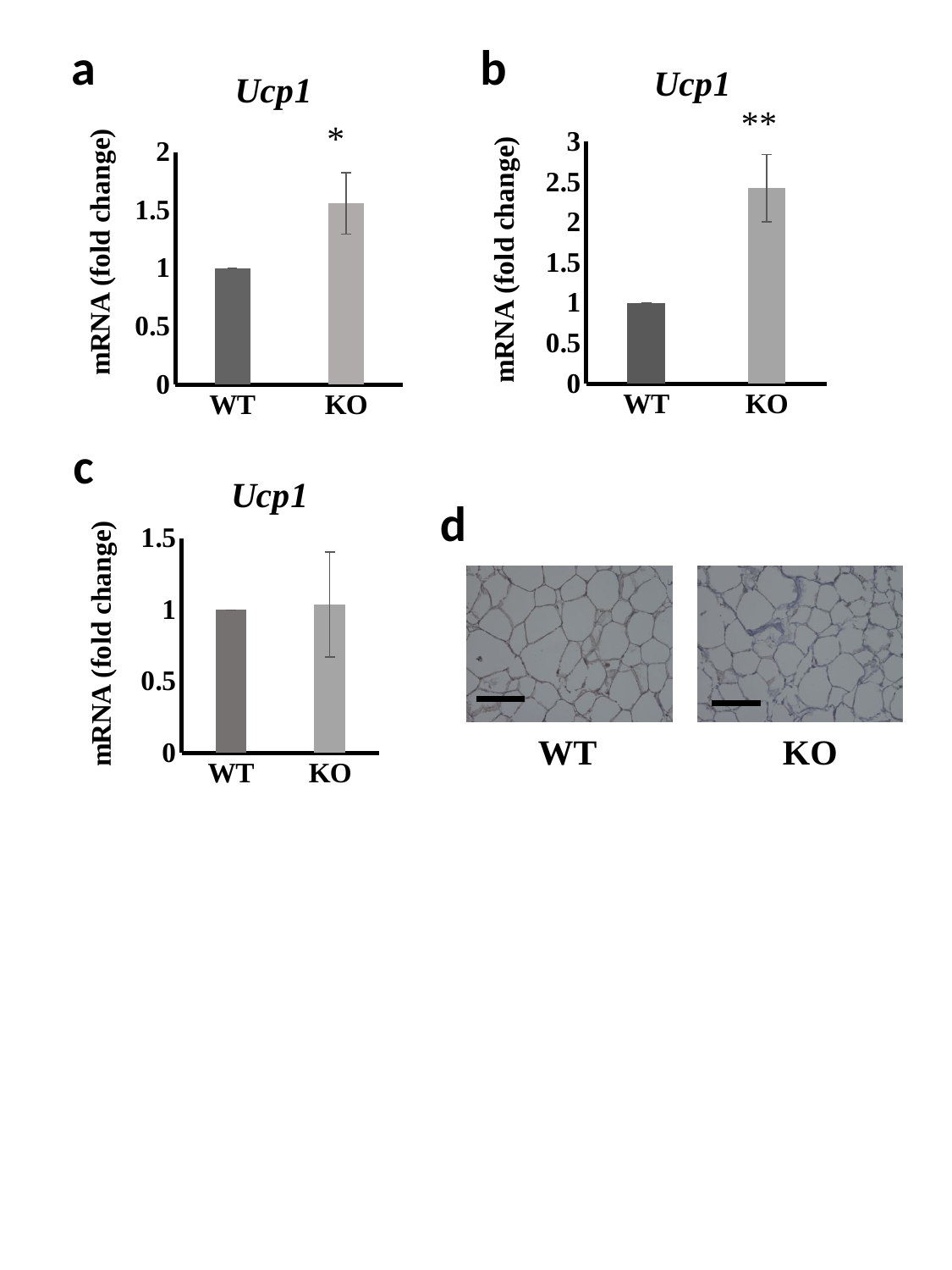

a
b
### Chart: Ucp1
| Category | |
|---|---|
| WT | 1.0 |
| KO | 2.4248100555291505 |
### Chart: Ucp1
| Category | |
|---|---|
| WT | 1.0 |
| KO | 1.5585264863351274 |c
### Chart: Ucp1
| Category | |
|---|---|
| WT | 1.0 |
| KO | 1.038446 |d
 WT KO

Supplement: Supplementary file 2 — Figure S2. (a) Ucp1 gene expression levels in BAT. (Data were normalized against β-actin levels.) (b) Ucp1 gene expression levels in eWAT. (Data were normalized against β-actin levels.) (c) Ucp1 gene expression levels in iWAT (Data were normalized against Gapdh levels). (d) Ucp1 staining in iWAT, bar: 100 μm. Brown parts are positively stained for Ucp1. (PPTX 591 kb) [file 12986_2019_372_MOESM2_ESM.pptx]
